# Interferon-induced ADP-ribosylation: technical developments driving ICAB discovery

**DOI:** 10.1042/BSR20240986

**Published:** 2025-03-14

**Authors:** Victoria Chaves Ribeiro, Lilian Cristina Russo, Dulce María González Duré, Nícolas Carlos Hoch

**Affiliations:** 1 Department of Biochemistry, Chemistry Institute, University of São Paulo, São Paulo, Brazil

**Keywords:** ADP-ribosylation, ICAB, innate immunity, interferon, PARP

## Abstract

Cells respond to a variety of internal and external stimuli by regulating the activities of different signalling cascades and cellular processes, often via chemical modifications of biological macromolecules that modulate their overall levels, biochemical activities or biophysical interactions. One such modification, termed ADP-ribosylation (ADPr), is emerging as an important player in the interferon (IFN) response, but the molecular targets and functions of ADP-ribosyltransferases within this core component of innate immunity still remains unclear. We and others have recently identified that stimulation of IFN signalling cascades promotes the formation of a novel cytosolic structure in human cells that is enriched in ADP-ribosyl modifications. Here, we propose to name these structures ‘interferon-induced cytosolic ADPr bodies’ (ICABs) and discuss their known components and potential functions. We also review methods to detect ICABs (and cellular ADPr in general) using a range of recently developed reagents. This lays the foundation for future studies aimed at elucidating the molecular functions of ICABs and ADPr in innate immune responses, which is a central unanswered question in the field.

## Introduction

ADP-ribosylation (ADPr) is a post-translational modification catalysed by ADP-ribosyltransferase (ART) enzymes that utilise NAD^+^ as a substrate to modify target proteins, or other macromolecules such as nucleic acids, with either mono-ADP-ribosylation (mono-ADPr [MAR]) or poly-ADP-ribosylation (poly-ADPr [PAR]) [1–3]. Protein ADPr is involved in several cellular processes, including DNA repair, transcriptional regulation, chromatin remodelling, vesicle trafficking, protein biosynthesis, cell death, and viral response [3–11]. Among the human ARTs, 17 are classified as PARPs (or ARTDs, for *ADP-ribosyltransferases diphtheria toxin-like*), named PARP1-4, PARP6-16, and tankyrases 1 and 2 (TNKS1/2; also known as PARP5a/5b) [3]. These proteins are intracellular (as opposed to *cholera toxin-like* ARTC enzymes) and share a conserved ART domain, usually in their C-terminal regions [12]. Most members of this family catalyse MAR, with the exception of PARP1, PARP2, TNKS1, and TNKS2, which can catalyse PAR, and PARP13, which lacks ADPr transferase activity [3]. PARP9, although inactive by itself, forms a heterodimer with the E3 ubiquitin ligase DTX3L, which has been demonstrated to catalyse the conjugation of ADP-ribose and other chemically related biomolecules with ubiquitin [13–17]. PARP1 is the founding member of the ART family, and its role in DNA damage signalling is the most extensively studied and best understood function of ADPr, while the molecular functions of other PARPs remain less well characterised [3].

ADPr occurs on several amino acids, including serine, tyrosine, glutamate, aspartate, cysteine, arginine, lysine, histidine, and asparagine, as well as on phosphate groups and bases of nucleic acids [18–26]. This modification can be recognised by several ADPr-binding domains (readers) and is also reversible through the action of various hydrolases (erasers) that are often specific to particular ADPr acceptor sites [2]. Interestingly, it was recently demonstrated that some PARPs, such as PARP9 and PARP14, have hydrolytic macrodomains themselves, indicating that they might promote both ADPr modification and removal in a regulated manner [27–30]. In addition to the human hydrolases, some clinically relevant viruses, including coronaviruses, encode ADPr hydrolases that reverse host ADPr, highlighting the critical role of this modification in host–pathogen interactions [9,30–33]. Indeed, several PARP genes are up-regulated by an antiviral signalling cascade termed the interferon (IFN) response, suggesting that these enzymes contribute to innate immune responses [9,31]. However, whether individual PARPs have completely separate molecular functions or whether they co-operate to orchestrate a restricted set of ADPr-dependent processes remains currently unclear [9,33,34].

Recently, important technical advances and the development of novel ADPr detection reagents have significantly facilitated the study and characterisation of this modification [35–40]. These developments have led to the detection of IFN-induced MAR in a punctate cytoplasmic pattern first described in [41], which we suggest here to be a novel cytoplasmic structure that we propose to name ‘interferon-induced cytosolic ADPr body’ (ICAB).

### IFN response and ADP-ribosylation

The innate immune system serves as the host’s first line of defence against both infectious and sterile insults [42–44]. This defence is initiated when pattern recognition receptors (PRRs) detect pathogen-associated molecular patterns from microbes, or damage-associated molecular patterns released by damaged or dying cells. These PRRs then activate signalling pathways that lead to inflammatory responses and programmed cell death [42,45].

A central mediator of the innate immune response is the family of autocrine and paracrine cytokines termed interferons (IFNs), which can be divided into three major types: type I IFNs, including human IFNα, IFNβ, IFNε, IFNk, and IFNω; type II IFN, comprised solely by IFNγ; and type III IFNs, which consist of multiple IFNλ subtypes [43,46,47]. These IFN types are genetically distinct and are recognised by different cellular receptors, yet all of them induce an antiviral state [43,47]. Importantly, IFN responses are not limited to antiviral defences, as they also play significant roles against other pathogens, in anti-tumour immunity and in immune regulation [47–49].

Type I IFNs (such as IFNα and IFNβ) can be produced by most cells in response to viral infection and their receptors are ubiquitously expressed in most nucleated cells, in line with a more systemic role of type I IFNs. Type II IFN (IFNγ) can only be synthesised by lymphocytes, such as T cells and natural killer cells, but most cell types express IFNγ receptors and can, thus, respond to it [44,46]. Type III IFNs (IFNλs) are mainly produced by epithelial cells but can also be secreted by macrophages, monocytes, and dendritic cells [50]. Type III IFN receptors are preferentially expressed on epithelial cells, corroborating the important role of these IFNs in mucosal barriers, such as the respiratory and gastrointestinal tracts [47,51].

The binding of IFNs to their cellular receptors activates receptor-associated intracellular kinases of the JAK family, which phosphorylate transcription factors of the STAT family [52]. Once activated, these transcription factors translocate to the nucleus and bind to interferon-stimulated response element or gamma-activated sequence promoter elements to induce the transcription of hundreds of genes known as interferon-stimulated genes (ISGs) [52]. These ISGs induce an antipathogenic cellular state by targeting distinct steps in the viral replication cycle such as viral entry, viral protein synthesis, viral genome replication, and viral trafficking [34,52], as well as modulating the activation of adaptive immune responses, for example, by increasing antigen presentation [53,54].

At least 8 of the 17 human PARP genes, namely PARPs 7, 8, 9, 10, 11, 12, 13, and 14, are IFN-stimulated genes, but the precise functions of each of these enzymes in IFN responses are still under intense investigation [9,34]. PARP13, also known as zinc finger antiviral protein and zinc finger CCCH-type containing, antiviral 1 (ZC3HAV1), is thought to promote IFN signalling by enhancing the activation of the PRR RIG-I, as well as targeting viral RNAs for degradation [9,55]. PARP12 has been shown to facilitate the activation of NF-κB-dependent transcription, to modulate intracellular trafficking of viral proteins, and to promote the degradation of some viral proteins [56–58]. Additionally, PARP14 was shown to enhance IFN-β production and the activation of ISGs [59], and to promote STAT6 binding to its target genes [60,61]. In macrophages, PARP14 was also shown to MARylate STAT1, reducing the transcriptional activation of ISGs, in contrast to the PARP9/DTX3L complex, that seemed to promote IFNγ-induced STAT1 phosphorylation [62]. The PARP9/DTX3L complex was also shown to promote ISG expression and to facilitate viral protein degradation [63]. PARP10 is thought to inhibit NEMO-dependent signalling, a central component of the IKK kinase complex required for the activation of NF-κB signalling [64]. Likewise, PARP7 was shown to supress IFN signalling, either by inhibiting the TBK1 kinase [65] or by repressing downstream IRF3/IRF7-dependent gene expression [66], both of which reduce IFN production. PARP11 reduces IFN receptor levels by stabilising β-TrCP, a ubiquitin ligase that targets the type I IFN receptor subunit IFNAR1 for proteasomal degradation [67]. Interestingly, PARPs 7, 9, 10, 12, 13, and 14 contain putative or confirmed RNA-binding domains, suggesting their involvement in RNA-related physiological processes, which could impact viral RNA sensing or their function/stability [9,12,28,34].

Interest in the roles of ADPr in IFN signalling was renewed during the COVID-19 pandemic, given that SARS-CoV-2 and other coronaviruses encode a macrodomain (Mac1) that hydrolyses ADP-ribose modifications and affects viral replication and pathogenesis, suggesting a previously underappreciated role for ADPr in antiviral defences [68–71]. Mac1 is a domain contained in non-structural protein 3 (nsp3), the largest non-structural protein encoded in coronavirus genomes, which is part of a complex that forms a pore through which the coronaviruses RNA is translocated out of the double-membrane vesicle in which it is transcribed [72–74]. Nsp3 contains several modular protein domains, which includes the hydrolytic Mac1, two additional catalytically inactive macrodomains (Mac2 and Mac3), a papain-like protease domain, a ubiquitin-like domain, a nucleic-acid-binding domain, transmembrane domains, and a Y domain [71,75]. Similarly, ADPr-hydrolysing macrodomains are also found in other viruses such as alphaviruses, hepatitis E virus, and rubella virus [9,70,71], indicating that ADPr hydrolysis is a recurrent strategy for viral immune evasion. However, the cellular or viral targets of host ARTs in this context and how hydrolysis of these modifications could benefit viral pathogenesis are currently not clear. Nonetheless, several large efforts to develop macrodomain inhibitors as novel antiviral drugs are under way [41,76–81].

### IFN-induced cytoplasmic ADPr bodies (ICABs)

**Figure 1:**
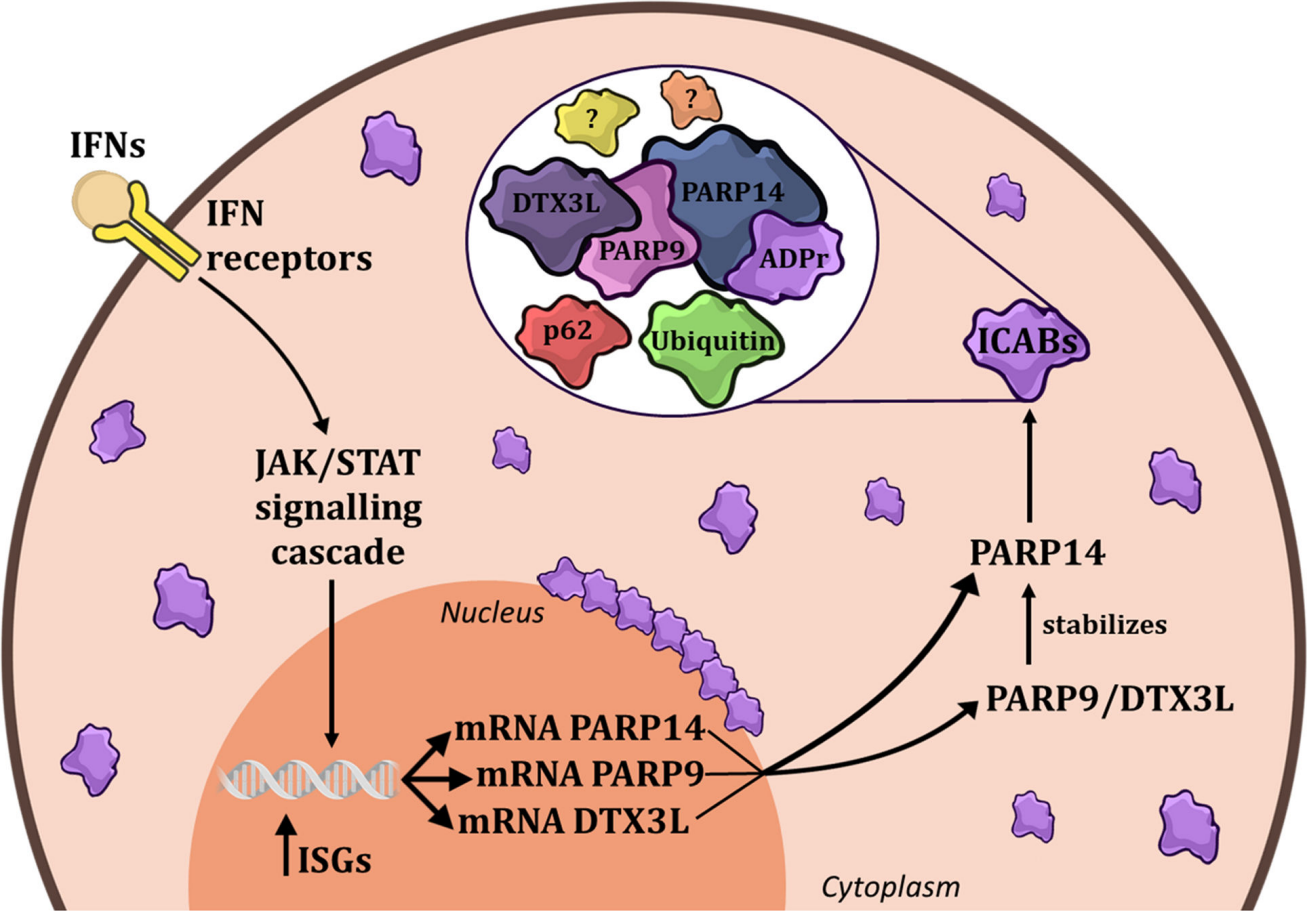
Components of interferon-induced cytosolic ADPr bodies (ICABs). In response to interferon signalling (either IFN-α, IFN-β, or IFN-γ), the JAK/STAT signalling cascade is activated, promoting the transcription of ISGs, including PARP14, PARP9, and DTX3L. The PARP9/DTX3L complex subsequently stabilizses PARP14 protein levels, which in turn catalyses mono-ADP-ribosylation within ICABs, composed of PARP14, the PARP9/DTX3L complex, ADPr, p62, ubiquitin, and other yet-to-be-identified components.

With the recent development of novel ADPr-binding reagents [35–37,39,82], it is now possible to detect cellular ADPr with unprecedented specificity and affinity. By using these tools to detect ADPr after IFN response stimulation of human cells, several groups have identified that IFN-induced ADPr is concentrated in a cytoplasmic structure of unknown function, which we propose to name ‘interferon-induced cytosolic ADPr body’ (ICAB) (Figure 1).

**Figure 2:**
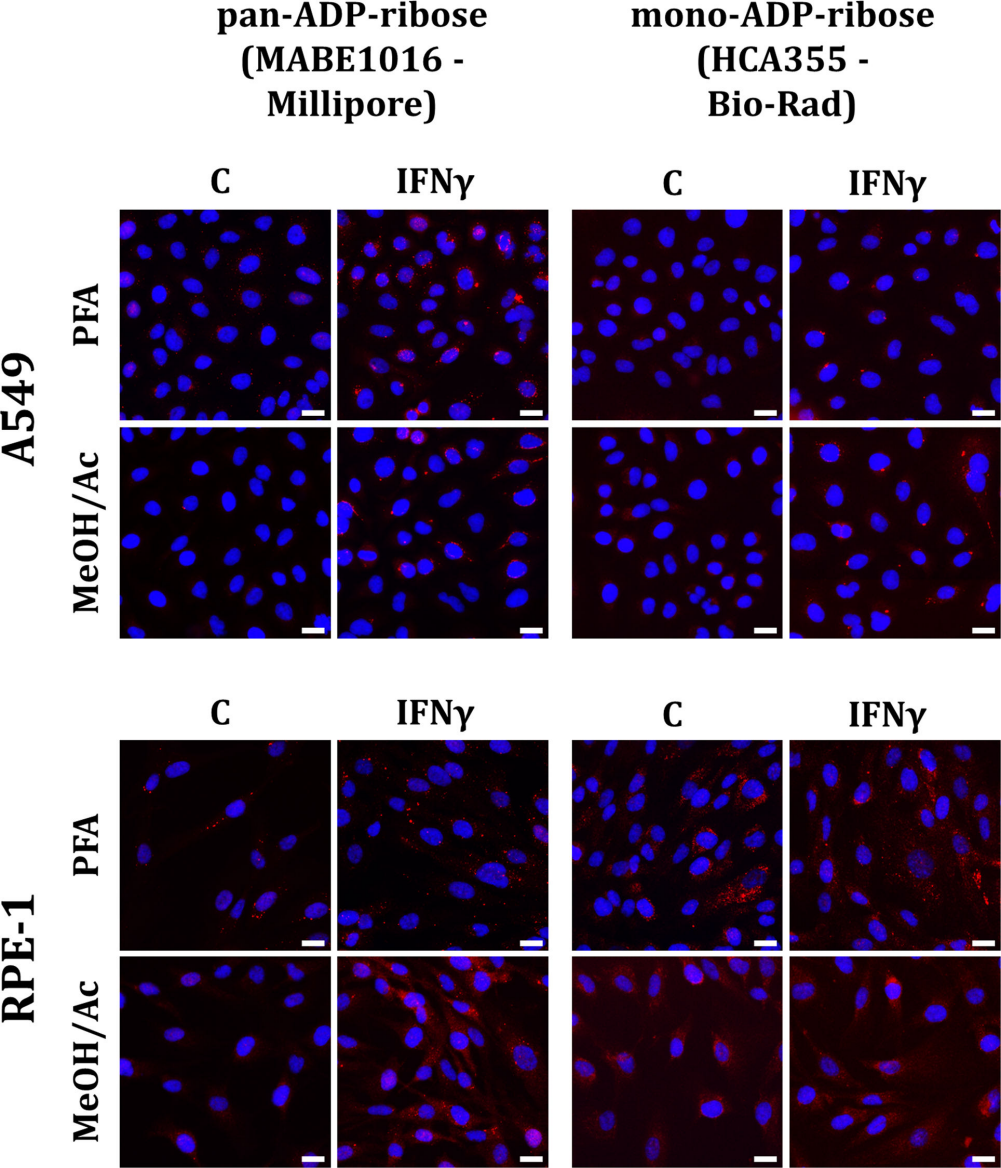
ICAB detection is directly influenced by cell line, fixation method, and detection reagent used. A549 and RPE-1 cells were treated with 200 U/mL IFNγ or vehicle control for 24 h and subjected to immunofluorescence using different combinations of fixation agent or ADPr detection reagent, in parallel samples processed on the same day. Cells were either fixed in 4% PFA for 10 min and permeabilised in 0.2% Triton X-100 in PBS minfor 10 min at RT or fixed/permeabilised in methanol/acetone for 10 min at 4°C. Samples were incubated with anti-pan-ADPr (MABE1016 Millipore) or anti-mono-ADPr (AbD33205/HCA355 -– Bio-Rad) and the same anti-rabbit AlexaFluor 568-conjugated secondary antibody. We observed higher intensity and lower dispersion of ICABs in A549 cells (upper) compared towith the RPE cell line (lower). Moreover, the labelling varied drastically depending on the reagent-fixation combination. Magnification: 20x. Red: ADP-ribose; blue: DAPI. Scale bar: 20 µm

ICABs were first reported by Russo et al. [41] as a cytosolic punctate ADP-ribose signal observed by immunofluorescence microscopy after treatment of human cells with either recombinant type I IFNs (IFNα and IFNβ) or type II IFN (IFNγ) or with poly(I:C), an RNA mimetic that induces a type I IFN signalling cascade. Interestingly, their formation is more evident after IFNγ treatment than in response to type I IFN signalling, which may indicate a more prominent function in type II IFN responses [41]. The formation of ICABs has been observed in several cell lines, including a lung carcinoma line (A549), hTERT-immortalised retinal pigment epithelial cells (RPE-1), HeLa cells, and a melanoma cell line (A375) [30,33,41,83,84]. ICABs can have different types of morphology, sometimes as a number of smaller structures that are closely spaced around the nucleus or in long cytoplasmic ‘lines’. Other times, ICABs present as a large number of small diffuse cytosolic puncta, or as a small number of very large cytosolic aggregates (Figure 2). It remains currently unclear whether these morphological differences are technical artefacts (discussed below) or represent biologically relevant differences between cell lines or even individual cells.

ICABs can be detected using several reagents (further discussed in the immunofluorescence section below) that detect either pan-ADP-ribose, without distinguishing mono- and poly-ADP-ribose, or reagents that recognise exclusively mono-ADP-ribose [33]. Additionally, the ADPr signal within ICABs is highly sensitive to hydroxylamine [33], a reagent that chemically removes ester-linked ADPr modifications, such as protein ADPr on acidic amino acids (aspartates and glutamates – D/E) [85,86]. These findings suggest that ICABs are enriched with proteins mono-ADP-ribosylated on D/E residues. Recent data have also identified IFN-induced modifications on cysteine residues [84], raising the possibility of a mixture of modification chemistries. However, given the technical difficulties in detecting ADPr on D/E residues (discussed below), it is possible that the D/E modifications may have been lost in the latter study.

The kinetics of ICAB induction, which start forming 6 h after treatment with recombinant IFNγ, reaching their peak at least 24 h post-treatment [41], indicated that this structure is not part of the initial IFN signalling cascade, but instead required induction of PARPs whose expression is controlled by IFN signalling. Indeed, inhibition of JAK kinases with tofacitinib prevented ICAB formation, whereas inhibition of PARP1/2 with olaparib did not [41]. Notably, either catalytic inhibition or silencing of PARP14 completely abolishes the IFN-induced ADPr signal within ICABs, suggesting a critical role of PARP14 activity for ICAB formation [30,33,83,84]. Additionally, the PARP9/DTX3L complex was also found to control ICAB formation, probably by regulating PARP14 activity; however, its precise molecular functions remain currently unclear. PARP14 protein levels are lower in PARP9/DTX3L-deficient cells [30,33,41,87] due to faster PARP14 protein turnover, which can be partially reversed by proteasome inhibition [33]. Interestingly, PARP14’s catalytic activity seems to play a role in this process, as PARP14 inhibition restores its protein levels in PARP9 or DTX3L knockout cells [33]. Together with biochemical data showing that DTX3L inhibits PARP14 activity and that the PARP9 macrodomain 1 can reverse PARP14-catalysed auto-ADPr [30], this suggests that the PARP9/DTX3L complex suppresses PARP14 activity and that PARP14 auto-ADPr promotes its degradation. However, this model does not explain why PARP9/DTX3L loss led to an apparent increase in IFN-induced ADPr by western blotting, despite a reduction in ICAB formation by immunofluorescence [30], or why PARP9 loss led to a milder effect on ICAB formation than DTX3L silencing/deletion [30,33], so further studies into the complex interplay between PARP9/DTX3L and PARP14 are required. The silencing of other IFN-induced PARPs – PARP7, PARP8, PARP10, PARP11, PARP12, and PARP13 – did not significantly affect the IFN-induced ADPr signal [33], indicating that PARP14 is the main enzyme responsible for catalysing IFN-induced MAR and is regulated by the PARP9/DTX3L complex (Figure 1).

Importantly, several proteins have already been localised to ICABs. Most notable among those are both PARP14 and the PARP9/DTX3L complex, again suggesting a critical role of these enzymes in promoting ADPr within ICABs [30,33,83,84]. Other components of ICABs include p62 (also known as Sequestosome 1; SQSTM1) and ubiquitin (Figure 1), which localise to some ICABs, but not all of them (Table 1) (30,83,84 and our own unpublished observations). Notably, it has been proposed that in response to IFN treatment, PARP14 auto-ADP-ribosylates and trans-ADP-ribosylates both DTX3L and p62 [28,30,33,83,84,88], indicating that these proteins are not only present in ICABs but are also the targets of ADPr. Interestingly, PARP14 was shown to ADP-ribosylate nucleic acids *in vitro*, including single-stranded RNA [12,28], which raises the intriguing possibility that nucleic acid ADPr may contribute to ICAB biology. Conspicuously, the PARP9/DTX3L complex was recently shown to catalyse the conjugation of ubiquitin onto a previously ADP-ribosylated peptide or nucleic acid *in* vitro [14,16], indicating that the sequential activity of PARP14 and the PARP9/DTX3L complex may promote the formation of hybrid ADPr-ubiquitin modifications within ICABs.

**Table 1:** Description of interferon-induced cytosolic ADPr bodies (ICABs) by different studies.

Reference	Cell lines/model	Treatments	Antibodies for detecting ADPr	Proteins/markers identified in ICABs
[41]	A549; RPE-1; HeLa	IFNγ; IFNα; IFNβ; Poly(I:C)	MABE1016	
[33]	A549; RPE-1	IFNγ	eAf1521; MABE1016; AbD33205 (HCA355); AbD33204 (HCA354)	PARP14, PARP9, DTX3L
[30]	A549	IFNγ	CST #83732; AbD43647 (TZA020)	PARP14, PARP9, ubiquitin
[83]	A549; A375	IFNγ	MABE1016; AbD33205 (HCA355)	p62, ubiquitin (FK2, K48-linked and K63-linked), NBR1, PARP14
[84]	A549	IFNβ	eAf1521	p62, PARP14, ubiquitin

Both p62 and poly-ubiquitin chains (which may be K48- or K63-linked [83]) associated with ICABs are key components of the main cellular protein degradation pathways. p62 acts as a scaffolding protein, recognising K48-polyubiquitinated proteins and directing them to proteasomal degradation. Additionally, p62 can also recognise K63-polyubiquitinated proteins, which promotes p62 oligomerisation and the formation of p62 bodies, subsequently recruiting the autophagy machinery [89]. Recent studies suggest that ICABs, unlike canonical p62 bodies, are insensitive to autophagy inhibition [83] and that the ADPr of p62 does not impair its autophagy function [84]. Moreover, while the interplay between ICABs and the ubiquitin–proteasome system remains unclear, an active ubiquitin–proteasome system appears to be necessary for ICAB formation [83].

It remains to be determined whether ICABs indeed represent a novel subcellular structure. However, given that ICABs do not seem to co-localise with a growing list of cellular compartments, such as mitochondria, lysosomes, endoplasmic reticulum, Golgi apparatus, P bodies, stress granules, autophagosomes, or the proteasome, and even their co-localisation with p62 and ubiquitin is partial (83,84 and our unpublished observations), we argue that the naming of this structure is warranted at this point, even if only for practical reasons, and could turn out to be a temporary solution until its full composition and function is known. The involvement of p62 and ubiquitin in these structures suggests that ICABs could be hubs for targeted protein degradation, which could play important roles in IFN responses, for instance by promoting the degradation of viral proteins. Indeed, DTX3L was shown to be required for the proteasomal degradation of a viral protease [63], although how ICAB formation contributes to this process remains currently unknown. PARP14 was also shown to modify PARP13 [28,88,90], which is a known restriction factor for several viral families [91,92], and ADPr within ICABs is sensitive to ectopic expression of the SARS-CoV-2 Nsp3 macrodomain (Mac1) [28,33,41], which indicate an important role for ICABs in antiviral responses and underscore the need for further research into the role of ICABs in host defence mechanisms.

### Detection of ICABs

After their initial identification in 2021 [41], ICABs have been detected by several groups, using a variety of different ADPr detection reagents and in different cell lines, attesting to the robustness of this finding (Table 1) [30,33,83,84]. However, we have repeatedly observed that different combinations of detection reagent, cell fixation method, and cell line can substantially affect results. In an attempt to harmonise the interpretation of results from different labs and to assist researchers to expand on these findings, we discuss some of the technical insights we have accrued in recent years. However, although some of the published data from other groups are included in this discussion, we caution that this description is naturally biased by our experience, and our understanding of ADPr detection methodologies is likely to continue to evolve.

#### ADPr detection reagents

In contrast to the anti-mono/poly-ADPr antibody (CST#83732), which has been used in the field for many years and was generated by standard immunisation of animals, many of the recently developed ADPr detection reagents are recombinant antibody-like proteins. These are composed of an ADPr-binding moiety and either a crystallisable fragment (Fc) derived from antibody heavy chains, such that these reagents are detected by standard secondary antibodies, or are fused to proteins that allow their direct detection, such as green fluorescent protein (GFP) or horseradish peroxidase (HRP). The MABE1016 pan-ADP-ribose reagent (Millipore) is composed of the *Archaeglobus fulgidus Af1521* macrodomain, which binds both MAR and PAR, fused to rabbit Fc, and was generated by the Kraus group in parallel with a similar MAR-specific reagent based on PARP14 macrodomains 2 and 3 (MABE1076), and a PAR-specific reagent derived from the RNF146-WWE domain (MABE1031) [35]. These reagents were recently engineered to replace the rabbit Fc portion with that from mouse or goat, broadening their applicability [82]. Similarly, the Hottiger group engineered an improved *Af1521* macrodomain variant (termed *eAf1521*) containing mutations that improve specificity and affinity of the *Af1521* domain, and fused this domain to mouse Fc [37]. Another variation of this strategy is the MacroGreen reagent, developed by the Schüler lab [93], in which a different set of mutations designed to improve the *Af1521* domain were identified and the domain was fused directly to GFP, for one-step detection of ADPr. Noteworthy, *Af1521*-based reagents have been shown to have ADPr hydrolase activity towards Glu/Asp modifications, especially when used at room temperature, which may affect their ability to detect ADPr modification on these residues [37]. A fundamentally different approach was employed by the Matic group, who enzymatically generated serine mono-ADP-ribosylated peptides and employed them for phage display screens and other assays that led to the development of synthetic antibodies with specificity for MAR, which are commercially available through Bio-Rad. The AbD33204 (HCA354) and AbD33205 (HCA355) reagents are human/rabbit antibody chimeras that bind MAR, but AbD33204 (HCA354) has a reduced affinity for serine-linked modifications over the AbD33205 (HCA355) reagent, although both can detect non-serine modifications [36,39]. AbD33204 (TZA019) and AbD33205 (TZA021) are variants in which the same MAR binding moieties are fused to SpyTag2, which enables attachment of a variety of detection modules, such as Fc´s from different species or HRP-conjugated modules, via SpyCatcher technology [94,95]. The AbD43647 moiety is an affinity-matured variant of AbD33204 that has higher affinity for MAR and is available in the SpyTag format (TZA020) or already fused to a 3x-HRP module for one-step western blotting (TZA020P).

Importantly, the use of pan-ADPr reagents that can detect both MAR and PAR, while useful in identifying new roles of ADPr in general, can impact the interpretation of results and should be complemented with experiments using sets of reagents that distinguish between MAR and PAR, to ascertain the nature of the detected ADPr modification.

#### Detection of ICABs by immunofluorescence

In our experience, ICAB detection is heavily influenced by sample preparation, especially the correct combination of cell line, fixation conditions, and ADPr detection reagent, each of which will be discussed below.

Regarding cell line, we consistently observe that ICABs are much more clearly induced and more pronounced in A549 cells (a lung adenocarcinoma cell line), irrespective of the ADPr detection reagent or fixation method used [33,41], when compared with other cell lines (Figure 2). In line with this, ICABs have been studied predominantly in A549 cells [30,33,41,83,84], although they are also observed in HeLa [33], A375 [83] and RPE-1-hTERT cells [33,41]. Whether this reflects a more pronounced induction of IFN signalling in these cells or a higher propensity for ICAB formation remains currently unclear. Related to this point, one important consideration is the sensitivity of a given cell line/cell type to the IFN-inducing treatment. For example, while treatment with recombinant IFNs is thought to induce IFN signalling in most cell types, epithelial cells generally require transfection of PRR agonists such as poly(I:C), whereas specialised immune cells such as macrophages can internalise PRR agonists and can, therefore, be treated with poly(I:C) directly in the growth medium [96].

The second important variable is fixation method. It is well established that the preservation of subcellular structures, as well as the detection of epitopes by a given antibody or reagent, can be heavily influenced by fixation method. Organic solvents such as methanol or acetone dehydrate the sample, leading to the denaturation of proteins. While this may help expose epitopes that are otherwise inaccessible, these solvents have the drawback of artificially solubilising some membrane-associated proteins or cellular structures. On the other hand, paraformaldehyde (PFA) and other cross-linking agents are generally thought to better preserve cellular architecture, but cross-linking can mask some antibody epitopes and may cross-link metabolites to proteins. This is the case with NADH, which is most abundant in mitochondria, and cross-reacts with some ADPr detection reagents, such that PFA fixation can lead to an artefactual mitochondrial signal in some combinations of cell line/fixation/detection reagent [38]. This can be mitigated using ADPr detection reagents that are not thought to bind to cross-linked NADH, or using solvent-based fixatives. To illustrate the impact of fixation method on ADPr detection, we conducted a side-by-side comparison of two different ADPr detection reagents, using two fixation methods (4% PFA or methanol/acetone), and two cell lines (A549 and RPE-1). While some cell line/ADPr reagent combinations were virtually insensitive to fixation method, other combinations were clearly affected (Figure 2).

We have extensively used and compared different ADPr detection reagents (MABE1016, eAf1521-Fc, AbD33204 [HCA354], AbD33205 [HCA355], and AbD33205 [TZA021]; conjugated via SpyCatcher technology to mouse Fc) in A549 and RPE-1 cells [33,41]. For A549 cells, we detected ICABs using any of these reagents, although some combinations of ADPr reagent/fixation condition were clearly superior to others (Figure 2). In these cells, we were even able to co-stain different ADPr reagents with each other (eAf1521-Fc vs. AbD33204/HCA354 or AbD33205/HCA355) [33]. However, we observed that in RPE-1 cells, some of these reagents detect a basal mitochondrial signal, which could reflect the NADH cross-linking artefact discussed above (Figure 2) [36,38,97]. However, the eAf1521 and AbD33205 (HCA355) reagents are not thought to recognise cross-linked NADH [38], and this signal persisted in methanol-fixed samples (Figure 2 and data not shown), indicating that it may represent a true mitochondrial ADPr signal [97]. Nonetheless, this signal can interfere with the visualisation and especially quantification of ICABs, and should be avoided when studying ICABs. We also made the curious observation that conjugation of ADPr detection reagents using SpyCatcher technology can affect their performance. While the commercially available AbD33205 (HCA355) reagent performed well in A549 cells, the same AbD33205 moiety conjugated to a mouse Fc was less efficient in our hands, although ICABs were still visible (data not shown).

Finally, it is important to highlight that adequate quantification of the ADP-ribose signal contained in ICABs is an important step in achieving robust results and elucidating the pathways that affect ICAB dynamics. To allow the quantification of ICABs in thousands of cells per condition, we employ a high-content automated fluorescence microscopy system, previously described in [41]. Using this system, many adjacent fields of view are acquired for each sample and the signal is analysed using a sophisticated analysis workflow described in [41]. It should be noted that occasionally some pan-ADPr reagents detect a nuclear signal, which is likely a result of spurious activation of nuclear ADPr transferases such as PARP1. For this reason, the nuclear signal is routinely excluded from our analysis to focus on detecting cytoplasmic ICABs only.

In summary, although the detection of ICABs by immunofluorescence is relatively straightforward, empirical optimisation of the best combination of cell line, fixation method, and detection reagent can significantly affect the results.

#### Detection of IFN-induced ADP-ribosylation by western blotting

In contrast to immunofluorescence, detection of IFN-induced ADPr by western blotting can be a challenge. One of the possible reasons for this discrepancy could be the relatively harsher sample preparation conditions for western blotting, including pH changes and high temperatures, which can affect the stability of some ADPr modifications during the procedure (discussed below). Alternatively, there is a possibility that IFN-induced ADPr is also present on RNA molecules, which would, in principle, be detectable by immunofluorescence but not by western blotting [38].

In response to DNA damage, PARP1 catalyses the formation of poly-ADPr chains, which are easily detectable by western blotting using a variety of reagents [36,98–100]. This occurs partially because PARP1 is a highly active enzyme and poly-ADPr chains can have multiple epitopes for antibody recognition, but also because this ADPr modification is thought to predominantly target serine residues, which occurs through a relatively stable O-glycosidic linkage [25,40,101]. In contrast, IFN-induced ADPr is thought to be less abundant, to occur in the form of MAR (i.e. one epitope per modification), and to be predominantly targeted to glutamate and aspartate residues via a labile ester linkage that is sensitive to pH and temperature [33,40,88,90,102,103]. While cysteine modification in response to IFN treatment has also been reported [84] and would be expected to be much more stable than modification of acidic residues, in our experience, IFN-induced ADPr is sensitive to hydroxylamine treatment [33] and very sensitive to small differences in sample preparation, indicating a labile modification on Glu/Asp residues. Therefore, while we provide some guidance and discuss some critical points, the field is still evolving and empirical adjustments of different protocols will likely continue until a reliable methodology is settled upon. Indeed, the importance of improving current protocols was recently highlighted by the detection of PARP1-dependent modification of acidic residues in response to DNA damage, which was made possible by careful optimisation of several steps in the western blotting procedure [40].

Sample preparation is probably the most critical factor for reproducible detection of IFN-induced ADPr – and glutamate/aspartate-linked ADPr in general. Different labs seem to have arrived at different working methodologies, but a common thread is clearly an effort to prevent modification loss during sample preparation steps. One critical adjustment is to perform the whole western blotting procedure at lower temperatures than usual, from sample preparation through electrophoresis and blotting. Cells can be lysed in RIPA buffer at 4°C supplemented with benzonase (an enzyme that degrades DNA and RNA) and PARP1/2 and PARG inhibitors (to prevent activation of these enzymes during lysis), but direct lysis in denaturing sample buffer is also possible [28,30,33,40,84]. Some groups denature proteins in standard sample buffers at no more than 70°C for 3–10 min [30,33], while other groups use a higher SDS concentration that seems to allow full protein denaturation at room temperature [40]. Another critical point is the composition (and particularly the pH) of the denaturing sample buffer. We routinely employ standard Laemmli buffer (pH 6.8), in which Glu/Asp modification is likely to be more stable than in more alkaline conditions [102]. Indeed, Longarini, E.J. and Matić, I. [40] reported that incubation of samples in NuPAGE sample buffer (pH 8.5) for as short as 30 min already affected Glu/Asp ADPr detection. Another interesting point to discuss here are freeze/thaw cycles. In our experience, we observed that using fresh samples (prepared in RIPA buffer at 4°C and immediately heated in Laemmli buffer at 70°C) results in a stronger signal, which was lost when parallel samples were frozen in RIPA buffer and later thawed and denatured. However, similar tests conducted by the Matic group indicate that up to two freeze-thaw cycles in 4% SDS lysis buffer did not negatively affect the final result [40].

**Figure 3:**
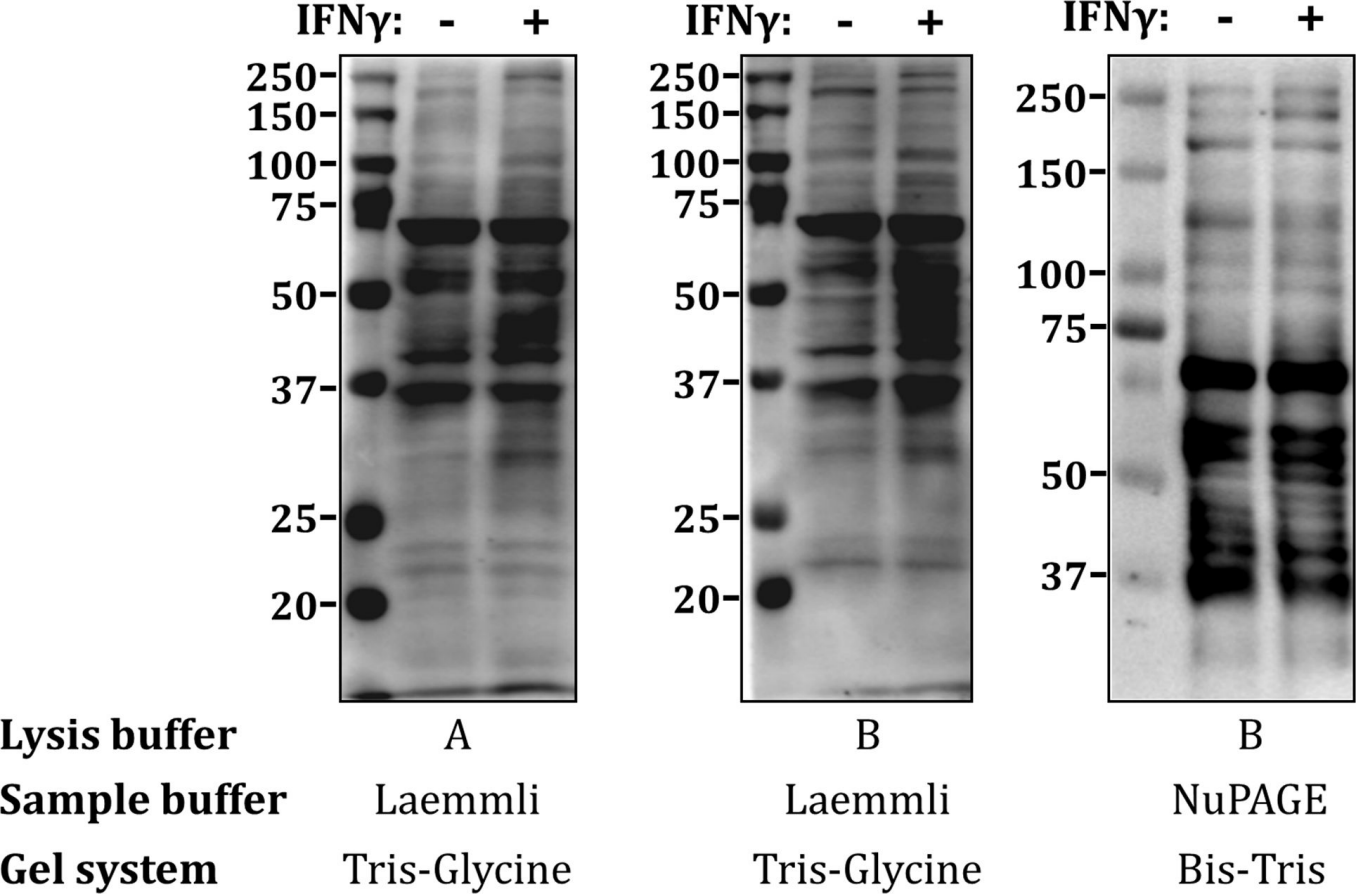
Detection of IFN-induced ADP-ribosylation by western blotting. Representative immunoblots using AbD43647 (TZA020P) for mono-ADPr detection in samples from A549 cells treated or not with 100 U/mL IFNγ for 24 h, processed by three different methods in parallel. Samples were collected in RIPA-based lysis buffers (buffer A: 100 mM Tris pH 7.5, 150 mM NaCl, 1% NP-40, 0.5% sodium deoxycholate, 0.1% SDS, 1 x protease inhibitor cocktail or buffer B: 50 mM Tris pH 8.0, 100 mM NaCl, 5 mM MgCl2, 1% Triton X-100, 1 x protease inhibitor cocktail, 2 µM olaparib, 1 µM PDD0017273, 250 U/mL benzonase [28]) and heated to 70°C for 10 min in Laemmli buffer (for Tris-Glycine gels) or in NuPAGE LDS buffer (for Bis-Tris gels), as indicated. All procedures were performed on ice or at 4°C.

While the sample preparation in pH 6.8 Laemmli buffer is likely preferable over the use of pH 8.5 NuPAGE LDS sample buffer, this is potentially compensated by the differences in subsequent gel electrophoresis conditions. Bis-Tris electrophoresis is performed at a more acidic pH of 6.5, compared with standard Tris-Glycine electrophoresis, which is pH 6.8 in the stacking gel, but as high as pH 8.8 in the resolving gel. However, we have achieved reproducible results using the conventional Laemmli buffer/Tris-Glycine combination [33] and have not observed a significant improvement when switching to the NuPAGE Bis-Tris system (Figure 3). As mentioned above, adequate temperature control during electrophoresis is extremely important, both by using chilled buffers and performing the electrophoresis and transfer procedures in a cold room, as well as by using lower voltages that prevent excessive buffer heating.

As already discussed for immunofluorescence above, the choice of ADPr detection reagent is also a crucial factor. While we have not comprehensively tested all commercially available antibodies, we were unable to detect IFN-induced ADPr by immunoblot with most reagents that perform well in immunofluorescence staining, such as pan-ADP-ribose (MABE1016 – Millipore), AbD33204 (HCA354) or AbD33205 (HCA355). On the other hand, we and others have obtained reproducible results using the AbD43647 antibody conjugated with 3xHRP (TZA020P – BioRad) or conjugated with the mouse Fc portion [28,30,33]. The eAF1521-Fc reagent has also been successfully used for the detection of IFN-induced ADPr by western blotting [84], although the latter study likely detected cysteine modification.

Rather than providing a fail-safe protocol (which seems to be still evolving in most groups), this discussion highlights how sensitive IFN-induced ADPr is to small methodological variations and how meticulous researchers need to be to establish a working protocol that allows reproducible and reliable results.

## Conclusions

The ADPr field has long been grappling with methodological difficulties in studying this modification, ranging from problems related to adequate detection of modification sites by mass spectrometry, the difficulty in producing antibodies that detect this modification with sufficient affinity and specificity (particularly for MAR) and the inherent chemical liability of some of the amino acid linkages. With the recent advent of different technologies and protocol adjustments to overcome these problems, which are still being improved upon, it became evident that IFN signalling induces the formation of a novel cytosolic structure containing ADPr-modified macromolecules, which we propose here to name ICABs. The elucidation of the molecular functions of ICABs in IFN signalling will be critical to help define the role of ADPr transferases in IFN responses, with important implications for our understanding of innate immune responses and the role of ADPr-hydrolysing macrodomains in viral pathogenesis.

## Abbreviations

ADPr: ADP-ribosylation
ART: ADP-ribosyltransferase
ICABs: interferon-induced cytosolic ADPr bodies
IFN: interferon
ISGs: interferon-stimulated genes
Mac1: macrodomain 1
Nsp3: non-structural protein 3
PRRs: pattern recognition receptors

## References

[1] Hoch N.C., Polo L.M (2020). ADP-ribosylation: from molecular mechanisms to human disease. Genet. Mol. Biol..

[2] Suskiewicz M.J., Prokhorova E., Rack J.G.M., Ahel I (2023). ADP-ribosylation from molecular mechanisms to therapeutic implications. Cell. Elsevier B.V.

[3] Lüscher B., Ahel I., Altmeyer M., Ashworth A., Bai P., Chang P. (2022). ADP-ribosyltransferases, an update on function and nomenclature. FEBS J..

[4] D’Amours D., Desnoyers S., D’Silva I., Poirier G.G (1999). Poly(ADP-ribosyl)ation reactions in the regulation of nuclear functions. Biochem. J..

[5] Mariotti L., Pollock K., Guettler S (2017). Regulation of Wnt/β-catenin signalling by tankyrase-dependent poly(ADP-ribosyl)ation and scaffolding. Br. J. Pharmacol..

[6] Leung A.K.L., Vyas S., Rood J.E., Bhutkar A., Sharp P.A., Chang P (2011). Poly(ADP-ribose) regulates stress responses and microRNA activity in the cytoplasm. Mol. Cell.

[7] Poirier G.G., de Murcia, G., Jongstra-Bilen J., Niedergang C., Mandel P (1982). Poly(ADP-ribosyl)ation of polynucleosomes causes relaxation of chromatin structure. Proc. Natl. Acad. Sci. U.S.A..

[8] Boamah E.K., Kotova E., Garabedian M., Jarnik M., Tulin A.V (2012). Poly(ADP-Ribose) polymerase 1 (PARP-1) regulates ribosomal biogenesis in Drosophila nucleoli. Plos Genet..

[9] Hoch N.C (2021). Host ADP-Ribosylation and the SARS-CoV-2 Macrodomain.

[10] Valente P.F., Hoch N.C (2024). Molecular mechanisms of cell death by parthanatos: more questions than answers. Genet. Mol. Biol..

[11] Grimaldi G., Corda D (2019). ADP-ribosylation and intracellular traffic: an emerging role for PARP enzymes. Biochem. Soc. Trans..

[12] Suskiewicz M.J., Munnur D., Strømland Ø., Yang J.C., Easton L.E., Chatrin C. (2023). Updated protein domain annotation of the PARP protein family sheds new light on biological function. Nucleic Acids Res..

[13] Yang C.S., Jividen K., Spencer A., Dworak N., Ni L., Oostdyk L.T. (2017). Ubiquitin modification by the E3 ligase/ADP-Ribosyltransferase Dtx3L/Parp9. Mol. Cell.

[14] Zhu K., Suskiewicz M.J., Chatrin C., Strømland Ø., Dorsey B.W., Aucagne V. (2024). DELTEX E3 ligases ubiquitylate ADP-ribosyl modification on nucleic acids. Nucleic Acids Res..

[15] Dearlove E.L., Chatrin C., Buetow L., Ahmed S.F., Schmidt T., Bushell M. (2024). DTX3L ubiquitin ligase ubiquitinates single-stranded nucleic acids. Elife.

[16] Zhu K., Suskiewicz M.J., Hloušek-Kasun A., Meudal H., Mikoč A., Aucagne V. (2022). DELTEX E3 ligases ubiquitylate ADP-ribosyl modification on protein substrates. Sci. Adv..

[17] Zhu K., Chatrin C., Suskiewicz M.J., Aucagne V., Foster B., Kessler B.M. (2024). Ubiquitylation of nucleic acids by DELTEX ubiquitin E3 ligase DTX3L. EMBO Rep..

[18] Ogata N., Ueda K., Kagamiyama H., Hayaishi O (1980). ADP-ribosylation of histone H1. Identification of glutamic acid residues 2, 14, and the COOH-terminal lysine residue as modification sites. J. Biol. Chem..

[19] Mcdonald L.J., Moss J (1994). Enzymatic and Nonenzymatic ADP-Ribosylation of Cysteine.

[20] Altmeyer M., Messner S., Hassa P.O., Fey M., Hottiger M.O (2009). Molecular mechanism of poly(ADP-ribosyl)ation by PARP1 and identification of lysine residues as ADP-ribose acceptor sites. Nucleic Acids Res..

[21] Leslie Pedrioli D.M., Leutert M., Bilan V., Nowak K., Gunasekera K., Ferrari E. (2018). Comprehensive ADP-ribosylome analysis identifies tyrosine as an ADP-ribose acceptor site. EMBO Rep..

[22] Suskiewicz M.J., Palazzo L., Hughes R., Ahel I (2021). Progress and outlook in studying the substrate specificities of PARPs and related enzymes. FEBS J..

[23] Weixler L., Schäringer K., Momoh J., Lüscher B., Feijs K.L.H., Zaja R (2021). ADP-Ribosylation of RNA and DNA: From in Vitro Characterization to in Vivo Function.

[24] Schuller M., Raggiaschi R., Mikolcevic P., Rack J.G.M., Ariza A., Zhang Y. (2023). Molecular basis for the reversible ADP-ribosylation of guanosine bases. Mol. Cell.

[25] Leidecker O., Bonfiglio J.J., Colby T., Zhang Q., Atanassov I., Zaja R. (2016). Serine is a new target residue for endogenous ADP-ribosylation on histones. Nat. Chem. Biol..

[26] Jankevicius G., Ariza A., Ahel M., Ahel I (2016). The toxin-antitoxin system DarTG catalyzes reversible ADP-ribosylation of DNA. Mol. Cell.

[27] Delgado-Rodriguez S.E., Ryan A.P., Daugherty M.D (2023). Recurrent loss of macrodomain activity in host immunity and viral proteins. Pathogens.

[28] Đukić N., Strømland Ø., Elsborg J.D., Munnur D., Zhu K., Schuller M. (2023). PARP14 is a PARP with both ADP-ribosyl transferase and hydrolase activities. Sci. Adv..

[29] Torretta A., Chatzicharalampous C., Ebenwaldner C., Schüler H (2023). PARP14 is a writer, reader, and eraser of mono-ADP-ribosylation. J. Biol. Chem..

[30] Kar P., Chatrin C., Đukić N., Suyari O., Schuller M., Zhu K. (2024). PARP14 and PARP9/DTX3L regulate interferon-induced ADP-ribosylation. EMBO J..

[31] Fehr A.R., Singh S.A., Kerr C.M., Mukai S., Higashi H., Aikawa M (2020). The Impact of PARPs and ADP-Ribosylation on Inflammation and Host-Pathogen Interactions.

[32] Lüscher B., Verheirstraeten M., Krieg S., Korn P (2022). Intracellular mono-ADP- ribosyltransferases at the host–virus interphase. Cellular and Molecular Life Sciences. Springer Science and Business Media Deutschland GmbH.

[33] Ribeiro V.C., Russo L.C., Hoch N.C (2024). PARP14 is regulated by the PARP9/DTX3L complex and promotes interferon γ-induced ADP-ribosylation. EMBO J..

[34] Biaesch K., Knapp S., Korn P (2023). IFN-induced PARPs—sensors of foreign nucleic acids?. Pathogens.

[35] Gibson B.A., Conrad L.B., Huang D., Kraus W.L (2017). Generation and characterization of recombinant antibody-like ADP-ribose binding proteins. Biochemistry.

[36] Longarini E.J., Dauben H., Locatelli C., Wondisford A.R., Smith R., Muench C. (2023). Modular antibodies reveal DNA damage-induced mono-ADP-ribosylation as a second wave of PARP1 signaling. Mol. Cell.

[37] Nowak K., Rosenthal F., Karlberg T., Bütepage M., Thorsell A.G., Dreier B. (2020). Engineering Af1521 improves ADP-ribose binding and identification of ADP-ribosylated proteins. Nat. Commun..

[38] Weixler L., Ikenga N.J., Voorneveld J., Aydin G., Bolte T.M., Momoh J. (2023). Protein and RNA ADP-ribosylation detection is influenced by sample preparation and reagents used. Life Sci. Alliance.

[39] Bonfiglio J.J., Leidecker O., Dauben H., Longarini E.J., Colby T., San Segundo-Acosta P. (2020). An HPF1/PARP1-based chemical biology strategy for exploring ADP-ribosylation. Cell.

[40] Longarini E.J., Matić I (2024). Preserving ester-linked modifications reveals glutamate and aspartate mono-ADP-ribosylation by PARP1 and its reversal by PARG. Nat. Commun..

[41] Russo L.C., Tomasin R., Matos I.A., Manucci A.C., Sowa S.T., Dale K. (2021). The SARS-CoV-2 Nsp3 macrodomain reverses PARP9/DTX3L-dependent ADP-ribosylation induced by interferon signaling. J. Biol. Chem..

[42] Kanneganti T.D (2020). Intracellular Innate Immune Receptors: Life inside the Cell.

[43] Mesev E.V., LeDesma R.A., Ploss A (2019). Decoding type I and III interferon signalling during viral infection. Nature Microbiology. Nature Publishing Group.

[44] Du Q., Miao Y., He W., Zheng H (2023). ADP-ribosylation in antiviral innate immune response. Pathogens.

[45] Diamond M.S., Kanneganti T.D (2022). Innate immunity: the first line of defense against SARS-CoV-2. Nat. Immunol..

[46] Casanova J.L., MacMicking J.D., Nathan C.F (2024). Interferon-**γ** and infectious diseases: lessons and prospects. Science.

[47] Lazear H.M., Schoggins J.W., Diamond M.S (2019). Shared and distinct functions of type I and type III interferons. Immunity. Cell Press.

[48] Castro F., Cardoso A.P., Gonçalves R.M., Serre K., Oliveira M.J (2018). Interferon-gamma at the crossroads of tumor immune surveillance or evasion. Front. Immunol..

[49] Lees J.R (2015). Interferon gamma in autoimmunity: a complicated player on a complex stage. Cytokine.

[50] Kotenko S.V., Rivera A., Parker D., Durbin J.E (2019). Type III IFNs: beyond antiviral protection. Semin. Immunol..

[51] Ye L., Schnepf D., Staeheli P (2019). Interferon-λ orchestrates innate and adaptive mucosal immune responses. Nat. Rev. Immunol..

[52] Schoggins J.W (2024). Interferon-stimulated genes: what do they all do. Annu. Rev. Virol..

[53] McNab F., Mayer-Barber K., Sher A., Wack A., O’Garra A (2015). Type I interferons in infectious disease. Nat. Rev. Immunol..

[54] Paschen A., Melero I., Ribas A (2022). Central role of the antigen-presentation and interferon-γ pathways in resistance to immune checkpoint blockade. Annu. Rev. Cancer Biol..

[55] Ficarelli M., Neil S.J.D., Swanson C.M (2021). Targeted restriction of viral gene expression and replication by the ZAP antiviral system. Annu. Rev. Virol..

[56] Welsby I., Hutin D., Gueydan C., Kruys V., Rongvaux A., Leo O (2014). PARP12, an interferon-stimulated gene involved in the control of protein translation and inflammation. J. Biol. Chem..

[57] Grimaldi G., Filograna A., Schembri L., Lo Monte M., Di Martino R., Pirozzi M. (2022). PKD-dependent PARP12-catalyzed mono-ADP-ribosylation of Golgin-97 is required for E-cadherin transport from golgi to plasma membrane. Proc. Natl. Acad. Sci. U.S.A..

[58] Li L., Zhao H., Liu P., Li C., Quanquin N., Ji X. (2018). *PARP12* suppresses Zika virus infection through PARP-dependent degradation of NS1 and NS3 viral proteins. Sci. Signal..

[59] Caprara G., Prosperini E., Piccolo V., Sigismondo G., Melacarne A., Cuomo A. (2018). PARP14 controls the nuclear accumulation of a subset of type I IFN-inducible proteins. J. Immunol..

[60] Goenka S., Cho S.H., Boothby M (2007). Collaborator of stat6 (CoaSt6)-associated poly(ADP-ribose) polymerase activity modulates Stat6-dependent gene transcription. J. Biol. Chem..

[61] Mehrotra P., Riley J.P., Patel R., Li F., Voss L., Goenka S (2011). PARP-14 functions as a transcriptional switch for Stat6-dependent gene activation. J. Biol. Chem..

[62] Iwata H., Goettsch C., Sharma A., Ricchiuto P., Goh W.W.B., Halu A. (2016). PARP9 and PARP14 cross-regulate macrophage activation via STAT1 ADP-ribosylation. Nat. Commun..

[63] Zhang Y., Mao D., Roswit W.T., Jin X., Patel A.C., Patel D.A. (2015). PARP9-DTX3L ubiquitin ligase targets host histone H2BJ and viral 3C protease to enhance interferon signaling and control viral infection. Nat. Immunol..

[64] Verheugd P., Forst A.H., Milke L., Herzog N., Feijs K.L.H., Kremmer E. (2013). Regulation of NF-κB signalling by the mono-ADP-ribosyltransferase ARTD10. Nat. Commun..

[65] Yamada T., Horimoto H., Kameyama T., Hayakawa S., Yamato H., Dazai M. (2016). Constitutive aryl hydrocarbon receptor signaling constrains type I interferon-mediated antiviral innate defense. Nat. Immunol..

[66] Manetsch P., Böhi F., Nowak K., Leslie Pedrioli D.M., Hottiger M.O (2023). PARP7-mediated ADP-ribosylation of FRA1 promotes cancer cell growth by repressing IRF1- and IRF3-dependent apoptosis. Proc. Natl. Acad. Sci. U.S.A..

[67] Guo T., Zuo Y., Qian L., Liu J., Yuan Y., Xu K. (2019). ADP-ribosyltransferase PARP11 modulates the interferon antiviral response by mono-ADP-ribosylating the ubiquitin E3 ligase β-TrCP. Nat. Microbiol..

[68] Grunewald M.E., Chen Y., Kuny C., Maejima T., Lease R., Ferraris D. (2019). The coronavirus macrodomain is required to prevent PARP-mediated inhibition of virus replication and enhancement of IFN expression. Plos Pathog..

[69] Voth L.S., O’Connor J.J., Kerr C.M., Doerger E., Schwarting N., Sperstad P. (2021). Unique mutations in the murine hepatitis virus macrodomain differentially attenuate virus replication, indicating multiple roles for the macrodomain in coronavirus replication. J. Virol..

[70] Alhammad Y.M., Parthasarathy S., Ghimire R., Kerr C.M., O’Connor J.J., Pfannenstiel J.J. (2023). SARS-CoV-2 Mac1 is required for IFN antagonism and efficient virus replication in cell culture and in mice. Proc. Natl. Acad. Sci. U.S.A..

[71] Alhammad Y.M.O., Kashipathy M.M., Roy A., Gagné J.P., McDonald P., Gao P. (2021). The SARS-CoV-2 conserved macrodomain is a mono-ADP-ribosylhydrolase. J. Virol..

[72] Huang Y., Wang T., Zhong L., Zhang W., Zhang Y., Yu X. (2024). Molecular architecture of coronavirus double-membrane vesicle pore complex. Nature.

[73] Chen A., Lupan A.M., Quek R.T., Stanciu S.G., Asaftei M., Stanciu G.A. (2024). A coronaviral pore-replicase complex links RNA synthesis and export from double-membrane vesicles. Sci. Adv..

[74] Wolff G., Limpens R.W.A.L., Zevenhoven-Dobbe J.C., Laugks U., Zheng S., de Jong A.W.M. (2020). A molecular pore spans the double membrane of the coronavirus replication organelle. Science.

[75] Lei J., Kusov Y., Hilgenfeld R (2018). Nsp3 of coronaviruses: structures and functions of a large multi-domain protein. Antiviral Res..

[76] Dasovich M., Zhuo J., Goodman J.A., Thomas A., McPherson R.L., Jayabalan A.K. (2022). High-throughput activity assay for screening inhibitors of the SARS-CoV-2 mac1 macrodomain. ACS Chem. Biol..

[77] Schuller M., Correy G.J., Gahbauer S., Fearon D., Wu T., Díaz R.E. (2021). Fragment binding to the Nsp3 macrodomain of SARS-CoV-2 identified through crystallographic screening and computational docking. Sci. Adv..

[78] Virdi R.S., Bavisotto R.V., Hopper N.C., Vuksanovic N., Melkonian T.R., Silvaggi N.R. (2020). Discovery of drug-like ligands for the mac1 domain of SARS-CoV-2 Nsp3. SLAS Discov..

[79] Sherrill L.M., Joya E.E., Walker A., Roy A., Alhammad Y.M., Atobatele M. (2022). Design, synthesis and evaluation of inhibitors of the SARS-CoV-2 nsp3 macrodomain. Bioorg. Med. Chem..

[80] Roy A., Alhammad Y.M., McDonald P., Johnson D.K., Zhuo J., Wazir S. (2022). Discovery of compounds that inhibit SARS-CoV-2 Mac1-ADP-ribose binding by high-throughput screening. Antiviral Res..

[81] Gahbauer S., Correy G.J., Schuller M., Ferla M.P., Doruk Y.U., Rachman M. (2023). Iterative computational design and crystallographic screening identifies potent inhibitors targeting the Nsp3 macrodomain of SARS-CoV-2. Proc. Natl. Acad. Sci. U.S.A..

[82] Chiu S.P., Camacho C.V., Kraus W.L (2024). Development and characterization of recombinant ADP-ribose binding reagents that allow simultaneous detection of mono and poly ADP-ribose. J. Biol. Chem..

[83] Raja R., Biswas B., Abraham R., Liu H., Chang C.Y., Vu H (2024). Interferon-Induced PARP14-Mediated ADP-Ribosylation in p62 Bodies Requires an Active Ubiquitin-Proteasome System. bioRxiv.

[84] Kubon D., Leslie Pedrioli D.M., Hottiger M.O (2024). PARP14 mediated SQSTM1/p62 cysteine ADP-ribosylation is counteracted by the SARS-CoV-2 macrodomain. bioRxiv.

[85] Burzio L.O., Riquelme P.T., Koide S.S (1979). ADP ribosylation of rat liver nucleosomal core histones. J. Biol. Chem..

[86] Moss J., Yost D.A., Stanley S.J (1983). Amino acid-specific ADP-ribosylation. J. Biol. Chem..

[87] Saleh H., Liloglou T., Rigden D.J., Parsons J.L., Grundy G.J (2024). KH-like domains in PARP9/DTX3L and PARP14 coordinate protein-protein interactions to promote cancer cell survival. J. Mol. Biol..

[88] Higashi H., Maejima T., Lee L.H., Yamazaki Y., Hottiger M.O., Singh S.A. (2019). A study into the ADP-ribosylome of IFN-γ-stimulated THP-1 human macrophage-like cells identifies ARTD8/PARP14 and ARTD9/PARP9 ADP-ribosylation. J. Proteome Res..

[89] Pohl C., Dikic I (2019). Cellular quality control by the ubiquitin-proteasome system and autophagy. Science.

[90] Carter-O’Connell I., Vermehren-Schmaedick A., Jin H., Morgan R.K., David L.L., Cohen M.S (2018). Combining chemical genetics with proximity-dependent labeling reveals cellular targets of poly(ADP-ribose) polymerase 14 (PARP14). ACS Chem. Biol..

[91] Schwerk J., Soveg F.W., Ryan A.P., Thomas K.R., Hatfield L.D., Ozarkar S. (2019). RNA-binding protein isoforms ZAP-S and ZAP-L have distinct antiviral and immune resolution functions. Nat. Immunol..

[92] Zhu Y., Chen G., Lv F., Wang X., Ji X., Xu Y. (2011). Zinc-finger antiviral protein inhibits HIV-1 infection by selectively targeting multiply spliced viral mRNAs for degradation. Proc. Natl. Acad. Sci. U.S.A..

[93] García-Saura A.G., Herzog L.K., Dantuma N.P., Schüler H (2021). MacroGreen, a simple tool for detection of ADP-ribosylated proteins. Commun. Biol..

[94] Hentrich C., Putyrski M., Hanuschka H., Preis W., Kellmann S.J., Wich M. (2024). Engineered reversible inhibition of SpyCatcher reactivity enables rapid generation of bispecific antibodies. Nat. Commun..

[95] Zakeri B., Fierer J.O., Celik E., Chittock E.C., Schwarz-Linek U., Moy V.T. (2012). Peptide tag forming a rapid covalent bond to a protein, through engineering a bacterial adhesin. Proc. Natl. Acad. Sci. U.S.A..

[96] Zhou Y., Guo M., Wang X., Li J., Wang Y., Ye L. (2013). TLR3 activation efficiency by high or low molecular mass poly I:C. Innate Immun..

[97] Hopp A.K., Teloni F., Bisceglie L., Gondrand C., Raith F., Nowak K. (2021). Mitochondrial NAD^+^ controls nuclear ARTD1-induced ADP-ribosylation. Mol. Cell.

[98] Hanzlikova H., Gittens W., Krejcikova K., Zeng Z., Caldecott K.W (2017). Overlapping roles for PARP1 and PARP2 in the recruitment of endogenous XRCC1 and PNKP into oxidized chromatin. Nucleic Acids Res..

[99] Palazzo L., Leidecker O., Prokhorova E., Dauben H., Matic I., Ahel I (2018). Serine is the major residue for ADP-ribosylation upon DNA damage. Elife.

[100] Gibbs-Seymour I., Fontana P., Rack J.G.M., Ahel I (2016). HPF1/C4orf27 Is a PARP-1-interacting protein that regulates PARP-1 ADP-ribosylation activity. Mol. Cell.

[101] Larsen S.C., Hendriks I.A., Lyon D., Jensen L.J., Nielsen M.L (2018). Systems-wide analysis of serine ADP-ribosylation reveals widespread occurrence and site-specific overlap with phosphorylation. Cell Rep..

[102] Tashiro K., Wijngaarden S., Mohapatra J., Rack J.G.M., Ahel I., Filippov D.V. (2023). Chemoenzymatic and synthetic approaches to investigate aspartate- and glutamate-ADP-ribosylation. J. Am. Chem. Soc..

[103] Wallace S.R., Chihab L.Y., Yamasaki M., Yoshinaga B.T., Torres Y.M., Rideaux D. (2021). Rapid analysis of ADP-ribosylation dynamics and site-specificity using TLC-MALDI. ACS Chem. Biol..

